# Comparative transcriptome profiling of high and low oil yielding *Santalum album* L

**DOI:** 10.1371/journal.pone.0252173

**Published:** 2022-04-28

**Authors:** Tanzeem Fatima, Rangachari Krishnan, Ashutosh Srivastava, Vageeshbabu S. Hanur, M. Srinivasa Rao

**Affiliations:** 1 Genetics and Tree Improvement Division, Institute of Wood Science and Technology, Bangalore, India; 2 Department of Computational and Data Sciences, Laboratory for Structural Biology and Biocomputing, Indian Institute of Science, Bangalore, India; 3 Department of Biotechnology, Indian Institute of Horticultural Research Hessarghatta, Bangalore, India; 4 Forest Development Corporation of Maharashtra Limited, Nagpur, India; Assam Agricultural University Faculty of Agriculture, INDIA

## Abstract

East Indian Sandalwood (*Santalum album* L.) is highly valued for its heartwood and its oil. There have been no efforts to comparative study of high and low oil yielding genetically identical sandalwood trees grown in similar climatic condition. Thus we intend to study a genome wide transcriptome analysis to identify the corresponding genes involved in high oil biosynthesis in *S*. *album*. In this study, 15 years old *S*. *album* (*Sa*SHc and *Sa*SLc) genotypes were targeted for analysis to understand the contribution of genetic background on high oil biosynthesis in *S*. *album*. A total of 28,959187 and 25,598869 raw PE reads were generated by the Illumina sequencing. 2.12 million and 1.811 million coding sequences were obtained in respective accessions. Based on the GO terms, functional classification of the CDS 21262, & 18113 were assigned into 26 functional groups of three GO categories; (4,168; 3,641) for biological process (5,758;4,971) cellular component and (5,108;4,441) for molecular functions. Total 41,900 and 36,571 genes were functionally annotated and KEGG pathways of the DEGs resulted 213 metabolic pathways. In this, 14 pathways were involved in secondary metabolites biosynthesis pathway in *S*. *album*. Among 237 cytochrome families, nine groups of cytochromes were participated in high oil biosynthesis. 16,665 differentially expressed genes were commonly detected in both the accessions (*Sa*Hc and *Sa*SLc). The results showed that 784 genes were upregulated and 339 genes were downregulated in *Sa*Hc whilst 635 upregulated 299 downregulated in *Sa*SLc *S*. *album*. RNA-Seq results were further validated by quantitative RT-PCR. Maximum Blast hits were found to be against *Vitis vinifera*. From this study, we have identified additional number of cytochrome family in high oil yielding sandalwood accessions (*Sa*Hc). The accessibility of a RNA-Seq for high oil yielding sandalwood accessions will have broader associations for the conservation and selection of superior elite samples/populations for further genetic improvement program.

## Introduction

East Indian Sandalwood (*Santalum album* L; Family; Santalaceae) is evergreen hemi-parasitic perennial tree. *S*. *album* trees are found in semi-arid regions from India to the South pacific and the northern coast of Australia besides the Hawaii islands [1]. The economic value of sandalwood depends on the quantity of heartwood and its essential oil extracted from the heartwood as well roots of the mature trees of santalum spps. [2–5]. It has been used for perfumery, cosmetics, pharmaceutical, religious and cultural purposes over centuries [6]. Indian government categorized *S*. *album* as one of 32 recognized medicinal plant [7]. The essential oil is very important trait, which is subjected to host species, soil type, climatic effects and elite germplasm [8–12]. However the limited oil yield of sandalwood restricts the demand of oil. The sandalwood oil formation is independent of heartwood growth and it was assumed that constant amount of oil being formed nevertheless of trees/heartwood growth, similar age of trees and with the smaller diameter heartwood consisting trees may tend to have greater percentage of oil. The quality of oil is largely defined by the percentage of different fragrant sesquiterpenes within the oil, especially α and β santalol [5]. Out of other santalum species, *S*. *album* is valued as a source of high content of oil as it has high level of α and β santalol and it shows low variability in oil composition across its natural range [13]. Due to international demand for sandalwood heartwood and its oil, over the recent times *S*. *album* has been considered as private investment to develop a sandalwood industry [14]. Excessive harvest, habitat destruction and lack of pest management system, global sandalwood resources are threated globally which indicated the large-scale shortage and escalation the market price of sandalwood products [4, 15, 16]. Realizing the sharp decline in the sandalwood population, the Karnataka and Tamil Nadu Forest department amended the sandalwood act in 2001 and 2002 and declared the private sandalwood growers himself an owner of the sandalwood as per the amended Act. Further, Govt. of Karnataka made an amendment on the sale of sandalwood through Forest department and Government, Departments to eliminate the clandestine trade and to encourage farmers to take cultivation of Sandalwood on commercial scale during the last few years [7]. Due to the amendment, many of the private organizations and farmers have started raising sandalwood cultivation on their private/farm lands. Since sandalwood plantation is long term high investment by the farmers and forest department, so it is essential to identify and supply superior quality planting material to optimize the high economic returns than their investment.

The breeding improvement is little due to its long generation time and lack of information about high oil yielding accessions/populations. Considering the constant increasing the global demand for sandalwood oil and genetic improvement purposes, the identification of factors regulating these qualitative and quantitative variations in oil is a critical issue. It was hypothesized that accumulation of sandalwood oil is a complex and dynamic process, which influenced by multiple genetic and environmental factors [17]. Candidate oil biosynthesizing genes, multiomics, trait associated mapping have been performed to investigate the mechanism of oil biosynthesis and accumulation. With the advancement of high throughput sequencing technology, several transcriptome profiling of studies have been carried out in sandalwood [18–22]. Although earlier studies showed that sandalwood oil biosynthesis pathways, identification of key oil biosynthesis genes (Cytochrome P450, Sesquisabinene synthases, and Sesquiterpene synthases), there are very few references available on transcriptomic oil biosynthesis regulation and accumulation. As such there are no any studies pertaining on transcriptomic regulation of sandalwood clones grown in identical environmental conditions. In this study, we performed comparative transcriptomic profiling of two identical accessions that differ significantly in oil content to understand the dynamic regulation of high and low oil accumulation. Understanding the high and low oil variants of the trees, as even a slight percentage improvement in sandalwood oil content will lead to significant value [23, 24]. Our results provide new insight for better understanding of how to achieve more sandalwood oil production by manipulation of core pathways and gene involved.

## Materials and methods

### Sampling site

The selection of *S*. *album* samples for transcriptome analysis was grounded on three factors [1] known age and [2] grown in identical environmental condition [3] diseased free trees. Therefore we selected 15 year old *S*. *album* trees grown in Institute of Wood Science and Technology (13.011160^o^N 77.570185^o^E) Bangalore Karnataka and collected samples in the month of August 8^th^ 2018.

### Sample collection

For oil estimation and RNA isolation, the wood samples were collected up to GBH at 1.37M by using conventional drilling increment borer (leaf materials were takes as a positive control in RNA extraction process). The four core samples (two replicates of each sample) were marked as transition zone, heartwood and sapwood and frozen into liquid nitrogen. The samples were immediately stored in dry ice box and shipped to the Eurofins laboratory. Before RNA extraction from the core samples, the oil quantity and quality was estimated by UV-spectrophotometer followed by GC-MS analysis. Based on the oil variability in terms of high and low oil-yielding (*Sa*SHc and *Sa*SLc) samples were selected for *De novo* transcriptome analysis (S1 Table).

### RNA isolation, cDNA library preparation and sequencing

The total RNA was extracted from transition zones of the selected cores samples by using modified CTAB and LiCl method [25, 26] and to validate the RNA quality and quantity, sandalwood leaves were used as a positive control. The quality of isolated RNA measured by UV spectrophotometer at 260/280and 260/230 nm wavelengths and 1% agarose gel electrophoresis followed by measuring RNA concentration using a 2100 Bioanalyzer (Agilent Technologies). The concentration of RNA was obtained in *Sa*SHc 1460.90 ng/*μ*l and in *Sa*SLc 12.65 ng/μl. The mRNA from the total RNA was extracted by using the poly-T attached magnetic beads, followed by fragmentation process. The cDNA library of *S*. *album* was constructed using 2 μL of total purified mRNA from each sample by using Illumina TruSeq stranded mRNA preparation kit. 1st strand cDNA conversion was carried out by using Superscript II and Act-D mix to facilitate RNA dependent synthesis and then second strand was synthesized by using second strand mix. The dscDNA was purified by using AMPure XP beads followed by A-tailing adapter ligation. The libraries were analyzed through 4200 TapeStation system (Agilent Technologies) by using high sensitivity D1000 screen tape. The Pairing end Illumina libraries were loaded on NextSeq500 for cluster generation and sequencing. Total two RNA libraries were generated with the paired end sequencing. To obtain high quality concordant reads the sequenced raw data were processed by Trimmomatic v0.38 [27]. In-house script (in python and R) software was used to remove adapters, ambiguous reads and low quality sequences and the high quality paired-end reads were used for *De novo* Transcriptome assembly. RNA-Seq data were produced in FASTQ format and the whole sequence reads archive (SRA) database has been deposited in NCBI under Biosample accession: SAMN1569426 SRA accession number: PRJNA648820.

### *De novo* transcriptome assembly, unigenes classification and functional annotation

Trinity *de novo* assembler (v2.5) [28] was used to assemble transcripts from pooled reads of the samples with a kmer_25 and minimum contig length value up to 200 bp. The assembled transcripts were then further clustered into unigenes covering >90% at the 5X reads by using CD-HIT-EST-4.5.4 software [29] for further downstream analysis. Coding sequences (open reading frames, ORFs) within the unigenes (default parameters, minimum of 100 amino acid sequence) were predicted by TransDecoder v5.0. The longest ORFs were then subjected to BLAST analysis against PSD, UniProt, SwissProt, TrEMBL, RefSeq, GenPept and PDB databases to obtain protein information resource (PIR) for the prediction of coding sequences by Blast2GO software program [30].

### Functional annotation

The functional annotation of genes was performed by DIAMOND (BLASTX compatible aligner) program software [31]. The functional identification of coding sequences in biological pathways of the respective sample reads was assigned to reference pathways in KEGG (Eukaryotic database). The output of KEGG analysis included KEGG orthology, corresponding enzyme commission (EC) numbers and metabolic pathways of predicted CDS by using KEGG automated annotation server KAAS (http://www.genome.jp/kaas-bin/kaas_main) [32].

### Differential gene expression analysis

The differential expressed genes (DEGs) were identified between the corresponding samples by implementing a negative binomial distribution model in DESeq package (v.1.22.1_http://www.huber.embl.de/users/anders/DESq) [33]. The combination for differential analysis was calculated as *Sa*SHc (high oil yielding) vs *Sa*SLc (low oil yielding) *S*. *album*. To analyze the differentially expressed genes, two software’s (heatmap, and Scatter plot) were used to predict upregulated and downregulated genes in *S*. *album*. A heat map was constructed by using the log-transformed and normalized value of genes based on Pearson uncentered distance and average linkage method. The most similar transcriptome profile calculated by a single linkage method, a heatmap were generated, correlating sample expression profiles into colours. The heatmap shows the level of gene expression and represented as log2 ratio of gene abundance between high and low oil yielding samples. An average linkage hierarchical cluster analysis was performed on top 50 differentially expressed genes using multiple experiments viewer (MeV v4.9.0) [34]. The colour represents the logarithmic intensity of the expressed genes. Relatively high expression values were showed in red (identical profiles) and low expression values were showed in green (the most different profiles). The scatter plot is used for representing the expression of genes in two distinct conditions of each sample combination i.e., high and low oil yielding clones. It helps to identify genes that are differentially expressed in one sample with respect to the corresponding samples. This allows the comparison of two values associated with genes. The vertical position of each gene in form the of dots represents its expression level in the high oil yielding samples while the horizontal position represents its expression level in the treated samples. Thus, genes that fall above the diagonal are over-expressed and gene that fall below the diagonal are under expressed as compared to their median expression level in experimental grouping of the experiment.

### Quantitative RT-PCR analysis

Quantitative Real Time PCR was performed by using SYBR Green PCR master mix kit in a stepOnePlus Real Time PCR system (Applied Biosystem by Life Technologies, USA) to validate the gene expression profiles identified by RNA-Seq results. The cDNA was amplified in a 20 μL volume including 10 μL SYBR green PCR master mix, 2 μL cDNA, 2 μL of primers (1 μLfor each forward and reverse) and 6 μL of distilled water. RT-PCR master mix (TaKaRa). Six previously identified sandalwood oil biosynthesizing genes [13, 35, 36] (*SaMTPS*, *SaFPPS*, *SaDSX*, *SaGGPS*, *SaGPS*, and *SaCYP450*) specific primers were predicted using by the online tool Primer3 version 0.4.0 and synthesized at (Eurofins India Pvt. Ltd). The sequence of primers with a melting temperature between 60–61 ^0^C and a PCR product range of 151–229 bp were listed in (S2 Table). qRT-PCR was performed with stepOne Real time PCR system (Applied Biosystems, Thermofisher Scientific). The qRT-PCR reaction systems were as follows: 95^0^ C for 20 s, followed by 40 cycles of 95 ^0^C for 5s, 60 ^0^C for 30s and 72 ^0^C 40 sec. Three replicates for each of the two biological replicates were performed. The transcript profiles were normalized using the reference housekeeping gene actin and the relative expression level of candidate genes were calculated with the 2^-^ΔΔct standard quantitative method [37]. To compare the RNA-Seq and qPCR results, a linear correlation was calculated using the log2 of the normalized expression values. The fluorescence data were collected and analyzed with Step One analysis software.

## Results

### Qualitative analysis of *S*. *album* oil

The selected core samples were quantitatively and qualitatively analyzed. The total oil percentage was found 4.96% and 0.93% for respective samples. Along with the oil content, α/β-santalol variation in *Sa*SHc 59.30/32.21 and in *Sa*SLc 49.52/26.60 was observed (S1 Table).

### Library construction and transcriptome sequencing

A total of 38,785326 (*Sa*SHc) and 35,94,4784 (*Sa*SLc) raw PE reads were generated from the Illumina sequencing of *S*. *album* (Table 1). After removing adapters containing >5% unknown nucleotide sequences, ambiguous reads and low quality reads (reads with more than 10% quality threshold (QV) <20 phred score) 28,959187 and 25,598869 were obtained to respective samples. The total clean bases for *Sa*SHc were 4.4 GB with 47.67% GC and 3.8 GB with 48.62% GC content for *Sa*SLc. 141,781 clean pair-end reads were assembled into pooled non-redundant putative transcripts with the mean length of 1,149 bp followed by N50: 2,044. The obtained transcript length ranged from 201 to 15,872 (S3 Table). The transcripts were assembled into 31,918 unigenes with the mean length and N50 length 1,739 2,272 respectively (S3 Table). Of the unigenes we found 11.85% (3,785) 200–500 bp in length, 19.06% (6,085) were 500–1000 bp in length, 36.28% (11,582) were 1000–2000 bp in length, 19.35% (6179) 2000–3000 bp in length, 8.42% (2688) 3000–4000 bp in length, 2.96% (946) 4000–5000 bp in length and 2.04% (653) exceeded 5000 bp (S3 Table). A total number of coding sequences (CDS) in pooled samples were found 2.271 million with total 2.810 billion bp. (S3 Table). Sample wise number of CDS was in *Sa*SHc and *Sa*SLc was 2.12 million and 1.811 million followed by total CDS base length 2.657 billion in *Sa*SHc and 2.307 billion (S3 Table).

**Table 1:** Summary of cDNA library, RNA-Seq and *de novo* sequence assembly of combined (*Sa*SHc and *Sa*SLc) *S*. *album*.

Description	*Sa*SHc	*Sa*SLc
cDNA library size (bp)	252–662	232–571
Average cDNA size (bp)	416	375
No of raw reads	32,959187	29,598869
No. of PE reads	2.99 billion	2.55 billion
Number of bases	435.67 billion	384.98 billion
Total data in GB	4.4	3.89

### Gene functional annotation and classification

**Fig 1:**
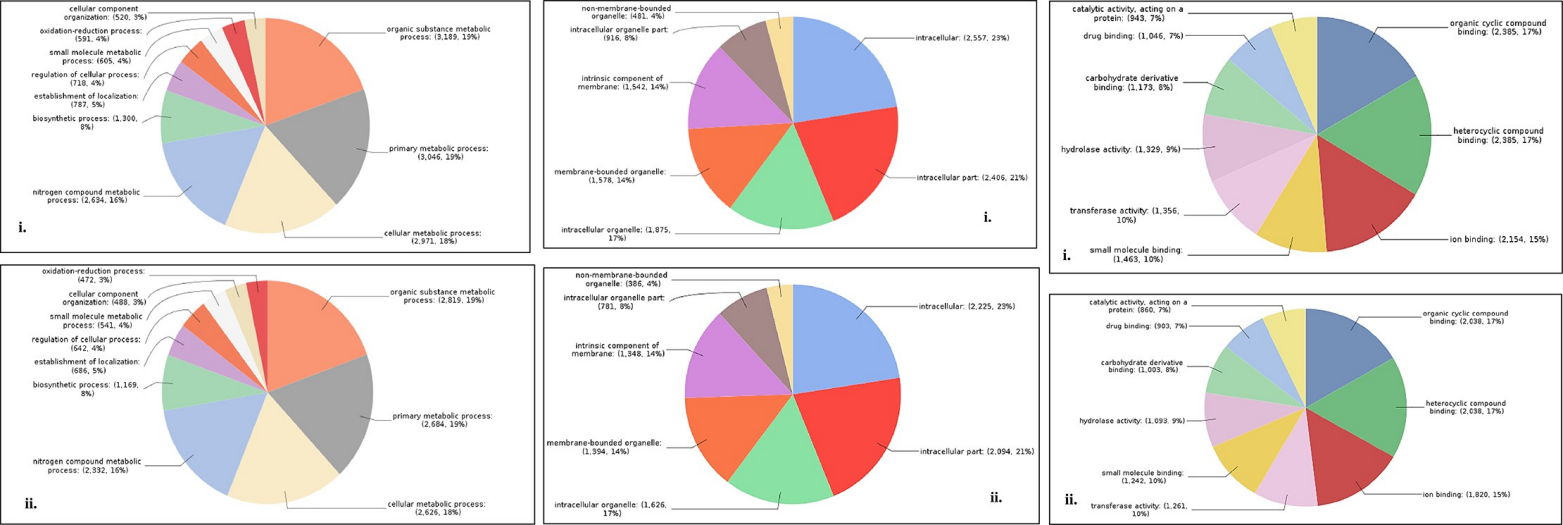
(**A).** Comparative GO biological regulation **i.** High oil yielding (*Sa*SHc) and **ii.** low oil yielding (*Sa*SHc) in *S*. *album*. **(B).** Comparative GO Cellular component **i.** High oil yielding (*Sa*SHc) and **ii.** low oil yielding (*Sa*SHc) in *S*. *album*. **(C).** Comparative GO Molecular function between **i.** High oil yielding (*Sa*SHc) and **ii.** low oil yielding (*Sa*SLc) in *S*. *album*.

**Fig 2:**
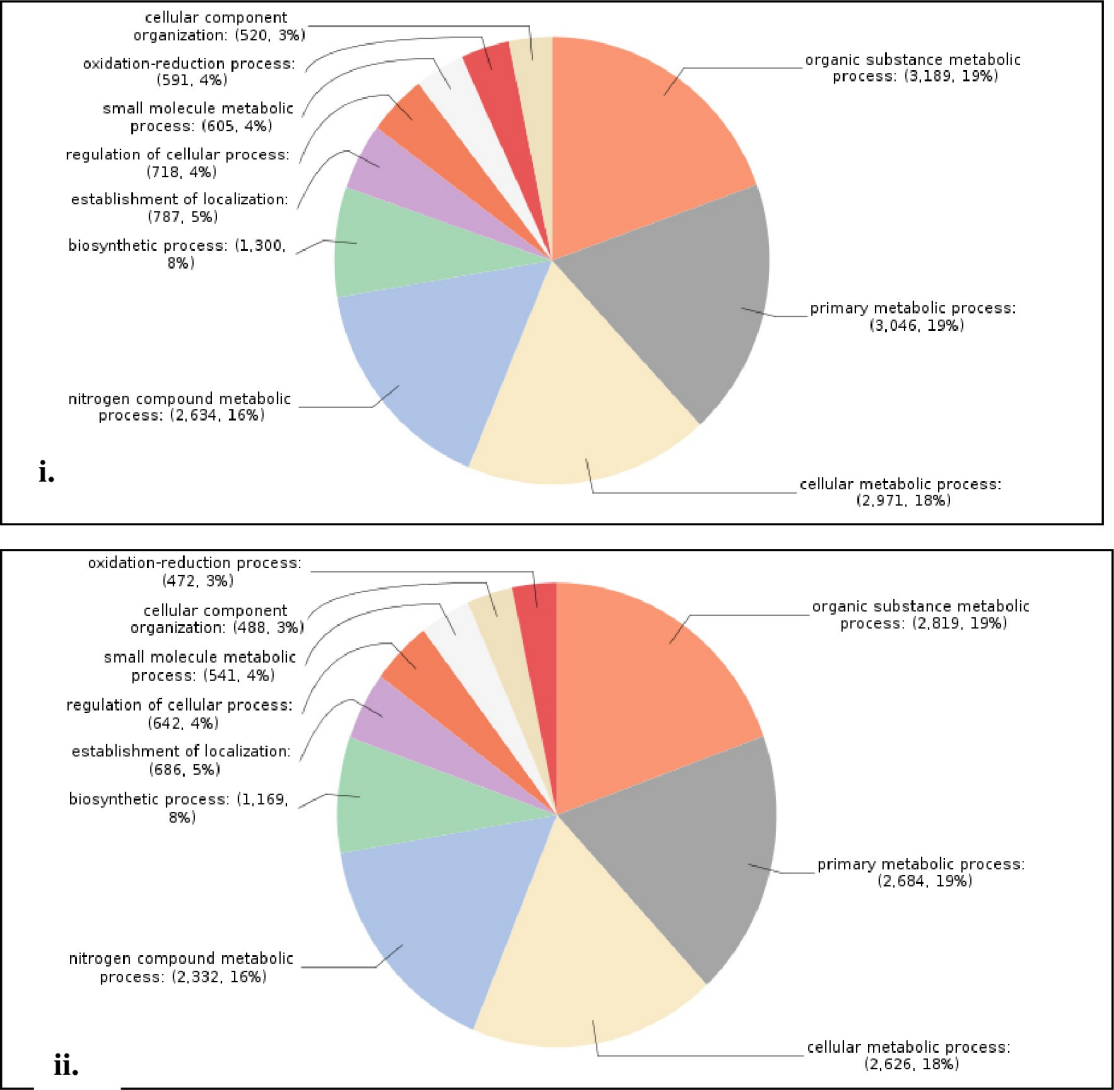
**(A).** Histogram of gene ontology classification (Wego plot); High oil yielding Sandalwood (*Sa*SHc). **(B).** Histogram of gene ontology classification (Wego plot); Low oil yielding Sandalwood (*Sa*SLc).

Total 22,710 CDS were BLAST and 20,842 CDS were annotated by NCBI databases (S3 Table). In case of *Sa*SHc and *Sa*SLc 20,262 and 18,113 genes were studied for Gene Ontology (GO). Based on the transcripts distribution, the assembled CDS were assigned into 26 functional groups of three GO categories: (i) Biological process (*Sa*SHc 4,168; *Sa*SLc 3,641) (ii) Molecular functions (*Sa*SHc 5108; *Sa*SLc 4,441) and (iii) Cellular components (*Sa*SHc 15,758; *Sa*SLc 4,971) (Table 2) (Fig 1A–1C). GO annotations for molecular functions (*Sa*SHc 13; *Sa*SLc 12), biological process (*Sa*SHc; 21, *Sa*SLc; 22) and cellular component analysis *Sa*SHc (16) and *Sa*SLc (17) were plotted by WEGO plotting tool. These domains were further containing Cellular component and in Molecular functions followed by Biological process respectively. The number of differential expressed genes (DEGs) in biological regulation terms was observed 5,108 in *Sa*SHc and 4,442 in *Sa*SLc. Data showed that prominent GO terms in biological process were metabolic process, cellular process, biological regulation, localization, stimulus, cellular component organization or biogenesis and signaling. Similar result was observed in cellular components *viz*, *Sa*SHc (4,168) and *Sa*SLc (3,642). In cellular components, majority of GO terms was related to cell, cell part organelle, membrane enclosed lumen, membrane and protein containing complex related genes was overrepresented in *Sa*SHc. In molecular function, the number of DEGs were involved in GO terms was 5,758 in *Sa*SHc and 4,972 in *Sa*SLc. The DEGs were prominently participated in catalytic activity, binding, transport activity, molecule carrier activity, antioxidant activity, and signal transducer activity. Among cellular components, cytosol, intracellular part, cytoplasmic fraction and cytoplasm were overrepresented in *Sa*SHc as compared to *Sa*SLc accessions. High number of genes was found in *Sa*SHc (41,900 genes) compared to *Sa*SLc (36,571 genes) that was further classified into biological process, cellular component and molecular functions. Highest number of genes was functionally annotated and was observed in biological process (*Sa*SHc 16,361) and (*Sa*SLc 14,459) followed by molecular function (Fig 2A and 2B).

**Table 2:** Samples wise Gene ontology (GO) category distribution of coding sequences (CDS) in *S*. *album*.

SI No.	Biological Process	Cellular Component	Molecular Function
***Sa*SHc**	5,108	4,168	5,758
***Sa*SLc**	4,441	3,641	4,971

### Kyoto encyclopedia of genes and genomes (KEGG) pathway mapping

Significant DEGs between *Sa*SHc and *Sa*SLc were mapped to reference canonical pathways in KEGG database. A total of 6,159 and 5,554 CDS of *Sa*SHc and *Sa*SLc were found to be categorized into 24 major KEGG pathways and were grouped in five main categories (Table 3). All assembled unigenes were subjected to further functional prediction and classification by KEGG Orthology (KO) database. Results showed 6,159 and 5,554 unigenes involvement in 24 groups in the KO database in respective samples and further subcategorized into 213 metabolic pathways (Table 3) (S1–S3 Figs). KEGG metabolite pathways represented 10 major pathways like metabolism, terpenoid synthesis, amino acid metabolism, purine metabolism, pyrimidine, transcription, translation, amino acyl-tRNA biosynthesis, DNA replication and membrane transport in sandalwood (Table 4). The EC numbers were classified in KEGG pathways, enabling the presentation of enzymatic functions in the context of the metabolic pathways. Among the identified pathways, secondary metabolite-flavonoid, and terpenoid related transcripts were over-represented (Table 4).

**Table 3:** Comparative KEGG pathway classification of coding sequences in high oil (*Sa*SHc) and low oil (*Sa*SLc) yielding *S*. *album*.

Pathways		*Sa*SHc		*Sa*SLc	
**Metabolism**					
Carbohydrate Metabolism		556		494	
Energy metabolism		323		281	
Lipid metabolism		272		231	
Nucleotide metabolism		162		147	
Amino acid metabolism		393		362	
Metabolism of other amino acids		156		138	
Glycan biosynthesis and metabolism		99		88	
Metabolism of cofactors and vitamins		218		193	
Metabolism of terpenoids and polyketides		99		80	
Biosynthesis of other secondary metabolites		86		77	
Xenobiotics biodegradation and metabolism		85		57	
**Environmental Information Processing**					
Membrane transport		34		30	
Signal transduction		597		645	
Signaling molecules and interaction		0		1	
**Cellular Processes**					
Transport and catabolism		458		426	
Cell growth and death		329		297	
Cellular community–eukaryotes		94		87	
Cellular community–prokaryotes		72		67	
Cell motility		51		44	
**Genetic information**					
Transcription		321		301	
Translation		739		652	
Folding, sorting and degradation		551		526	
Replication and repair		151		126	
**Organismal system**					
Environmental adaptation		264		253	

**Table 4:** Top 10 KEGG pathways mapped in sandalwood (*S*. *album*) transcripts.

SI No	KEGG pathways	*Sa*SHc	*Sa*SLc
1.	Metabolism	2981	2633
2.	Terpenoid synthesis	216	181
3.	Amino acid metabolism	557	495
4.	Purine metabolism	130	119
5.	Pyrimidine metabolism	82	73
6.	Transcription	321	301
7.	Translation	303	234
8.	Amino acyl tRNA biosynthesis	46	42
9.	DNA replication	27	26
10.	Membrane transport	34	30

### DEGs involved in sandalwood oil biosynthesis in *S*. *album*

DEGs were further annotated with KEGG database to deep insight the gene products for metabolism and functions related genes in different classified pathways. We performed an enrichment analysis of gene ontology (GO) terms with high significance in the upregulated DEGs. To identify metabolic pathways, *Sa*SHc (297) and *Sa*SLc (259) DEGs were mapped. As a result, 14 major pathways have been shown to play important role in sandalwood oil biosynthesis. Most pathways were resulted to secondary metabolites biosynthesis and metabolism by cytochrome P450. In order to identify secondary metabolite biosynthesis pathways in sandalwood, 4,697 transcripts for *Sa*SHc and 4,134 for *Sa*SLc were plotted. In Terpenoid backbone biosynthesis (35;33), Monoterpenoid biosynthesis (2;1), Sesquiterpenoid and Tri-terpenoid biosynthesis (4;3), Diterpenoid biosynthesis (10;10), Polyprenoid biosynthesis (31;30), Flavone and Flavanol biosynthesis (3;2), Isoquinolene alkaloid biosynthesis (9;6), Stilbenoid diaryl-heptanoid and Gingerol biosynthesis (3;4), Tropane piperidine and pyridine alkaloid biosynthesis (11;18) and Carotenoid biosynthesis (21;15) genes were involved in *Sa*SHc and *Sa*SLc sandalwood accessions. Predominantly genes were involved in metabolism of xenobiotics by Cytochrome P450 (*Sa*SHc 34; *Sa*SLc 23) and leads to up-regulation metabolic pathways. All these Go terms can be connected with sandalwood oil biosynthesis through an enhanced production of gene products in *S*. *album* oil biosynthesis pathway (Table 5).

**Table 5:** Comparative analysis of DEGs involved in secondary metabolite biosynthesis pathway analysis of Kos in high oil (*Sa*SHc) and low oil (*Sa*SLc) yielding sandalwood (*S*. *album*).

Pathway	Kos		Pathway ID
	*Sa*SHc	*Sa*SLc	
Terpenoid backbone biosynthesis	35	33	Ko00900
Monoterpenoid biosynthesis	2	1	Ko00902
Sesquiterpenoid and triterpenoid biosynthesis	4	3	Ko00909
Diterpenoid biosynthesis	10	10	Ko00904
Polyprenoid biosynthesis	31	30	Ko00940
Flavone and flavanol biosynthesis	3	2	Ko00944
Isoquinolene alkaloid biosynthesis	9	6	Ko00950
Drug metabolism: Cytochrome	31	23	Ko00982
Metabolism of xenobiotics by Cytochrome P450	34	23	Ko00980
Stilbenoid diarylheptanoid and gingerol biosynthesis	3	4	Ko00945
Tropane piperidine and pyridine alkaloid biosynthesis	11	8	Ko00960
Carotenoid biosynthesis	21	15	Ko00906
**Total**	194	158	

### Profiling of differential expressed genes (DEGs) participated in sandalwood oil biosynthesis regulation

**Fig 3:**
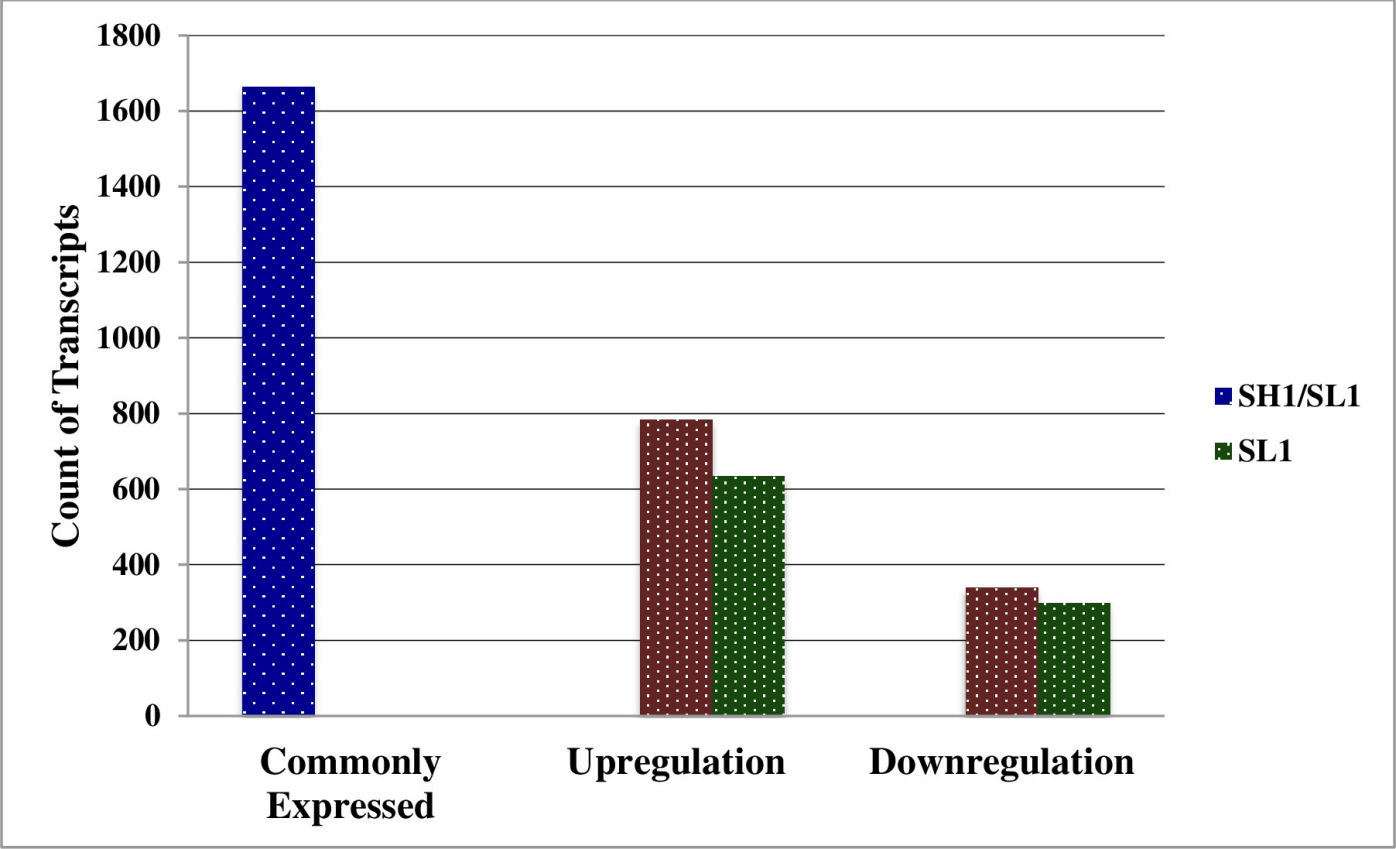
Identification of differentially expressed genes (DEGs) between *Sa*SHc and *Sa*SLc. Green Bar indicates commonly expressed DEGs. Blue and red bars represent upregulated and downregulated DEGs (significant at P-value threshold of 0.05).

**Fig 4:**
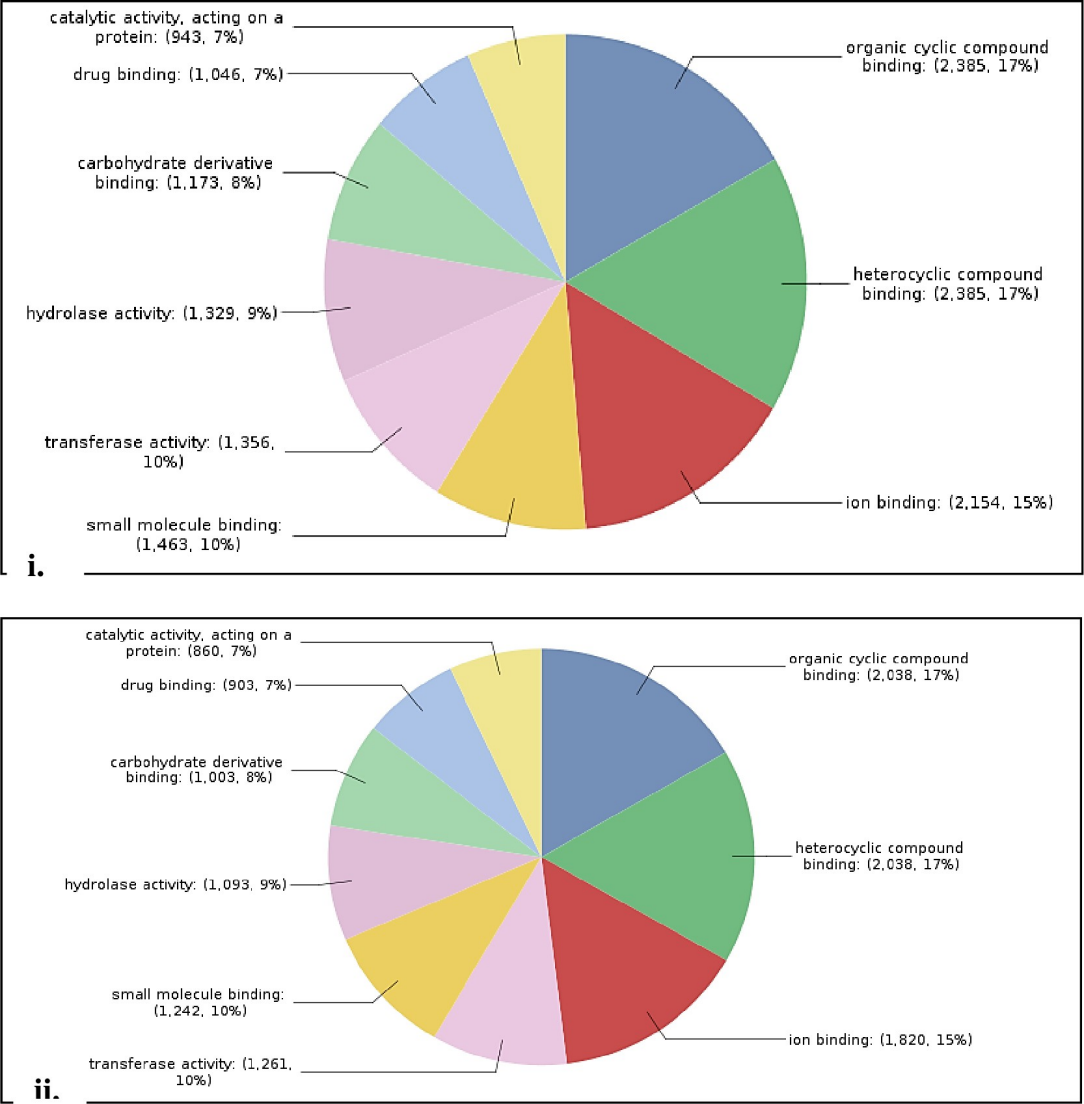
Visualization of differentially expressed gene transcription by (A) Scatter plot of differentially expressed genes between *Sa*SHc and *Sa*SLc (significant at P value <0.05); green dots represent the downregulated (significant) and red dot represents the upregulated (significant) genes for DGE combination (B) Volcano plot of differentially expressed genes; green dots represent the downregulated (significant) and red dot represents the upregulated (significant)genes for DGE combination (significant at P value <0.05).

**Fig 5:**
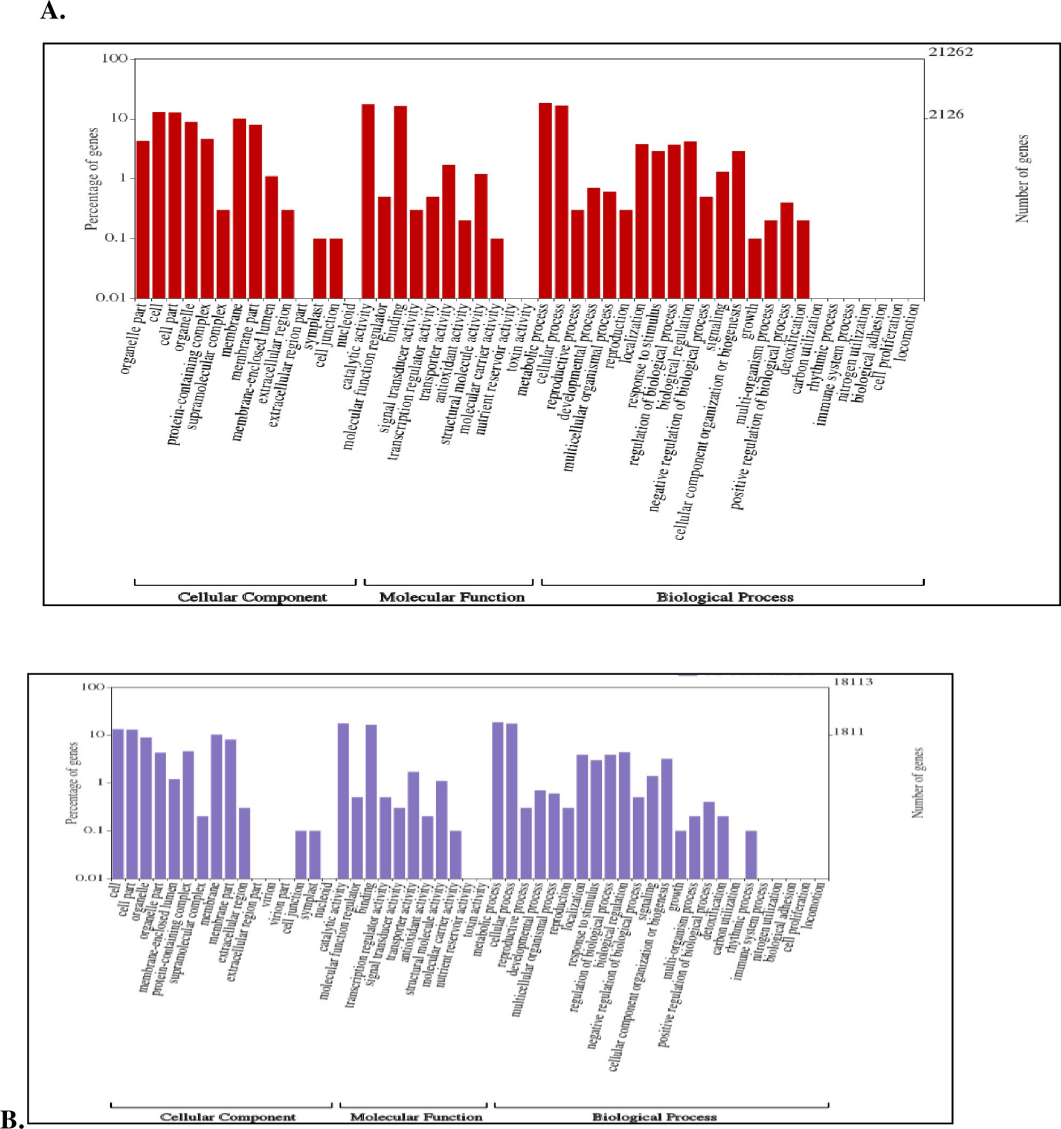
Heat map depicting the top 50 differentially expressed genes (significant); base mean *Sa*SHc represents the normalized expression values for *Sa*SHc sample and base mean and *Sa*SLc represents the normalized expression values for DGE combination.

All stages of sandalwood oil biosynthesis were examined, and a comparative analysis was done using aligned reads and the transcripts were grouped based on their degree of expression (log2FC). 16,665 differentially expressed genes were commonly detected in both the accessions (*Sa*Hc and *Sa*SLc). The results showed that 784 genes were upregulated and 339 genes were downregulated in high oil yielding accessions whilst 635 upregulated 299 downregulated in low oil yielding *S*. *album* accessions (Fig 3). Total biological process associated with DEGs (9 upregulated and 7 downregulated) in *Sa*SHc and *Sa*SLc sandalwood accessions were identified in which highly upregulated genes were identified in *Sa*SHc energy metabolism followed by secondary metabolite biosynthesis (Table 6). Genes related to oil biosynthesis in *S*. *album* have been commonly expressed and previously listed in gene expression pattern represented by Scatter plot showed a significant log 2FC>16.0; P value <0.005 for upregulated genes and log 2FC<0.40; P value <0.005 downregulated in case of *Sa*SHc sample (Table 7). Approximately 4.39% genes were found upregulated and 1.87% was downregulated in total differentially expressed genes. The normalized gene expression values from both the samples were used to estimate a Euclidian distance matrix based on transcript describing the similarities between the *Sa*SHc and *Sa*SLc samples. The red dots represented the upregulated genes and green dots represented the down regulated in DGE combination (Fig 4). Similar to scatter plot, based on their degree of expression (log2FC) values, Heatmap were also used to generate DEGs pattern. Heatmap showed transcript abundance level and indicated a similarity gradient between the *Sa*SHc and *Sa*SLc accessions. In Heatmap, gene expression was calculated in accordance with the method of FPKM, which takes into account the influence of both the sequencing depth and gene length on read count. In the FPKM distribution for selected samples, *Sa*SHc showed the highest probability density distribution of gene expression, whereas, *Sa*SLc displayed the lowest (Fig 5). The transcripts, which were highly expressed, were annotated for each gene as a high number of fold change and measure primarily the relative change of expression level. The top 50 highly upregulated genes (log2 FC 4.65–9.285) were shown in the Heatmap (Fig 5).

**Table 6:** Total biological process associated with differentially expresses genes (DEGs) in high and low oil yielding sandalwood (*S*. *album*).

Up regulated genes			
		*Sa*SHc	*Sa*SLc
**1.**	**Carbohydrate metabolism**	-	Glyoxylate and dicarboxylate metabolism [Pathway ID:ko00630]
**2.**	**Energy metabolism**	Sulfur metabolism [Pathway ID:ko00920]	-
		Cutin, suberine and wax biosynthesis [Pathway ID:ko00073]	-
		Steroid biosynthesis [Pathway ID:ko00100]	-
		Glycerolipid metabolism [Pathway ID:ko00561]	-
		Glycerophospholipid metabolism [Pathway ID:ko00564]	-
		-	Carbon fixation in photosynthetic organisms [Pathway ID:ko00710]
**3.**	**Lipid metabolism**	-	Fatty acid biosynthesis [Pathway ID:ko00061]
		-	
		-	Steroid biosynthesis [Pathway ID:ko00100]
		Sulfur metabolism [Pathway ID:ko00920] [Input number-1]	-
**4.**	**Nucleotide metabolism**	-	Purine metabolism [Pathway ID:ko00230]
**5.**	**Amino acid metabolism**	-	Cysteine and methionine metabolism [Pathway ID:ko00270]
		-	Arginine and proline metabolism [Pathway ID:ko00330]
		-	Tyrosine metabolism [Pathway ID:ko00350]
		-	Phenylalanine metabolism [Pathway ID:ko00360]
		-	Phenylalanine, tyrosine and tryptophan biosynthesis [Pathway ID:ko00400]
**6.**	**Metabolism of cofactors and vitamins**	Thiamine metabolism [Pathway ID:ko00730]	-
		Folate biosynthesis [Pathway ID:ko00790]	-
**7.**	**Biosynthesis of other secondary metabolites**	Flavonoid biosynthesis [Pathway ID:ko00941]	-
		Flavone and flavonol biosynthesis [Pathway ID:ko00944]	-
		Isoquinoline alkaloid biosynthesis [Pathway ID:ko00950]	-
**8.**	**Metabolism of terpenoids and polyketides**	Biosynthesis of siderophore group nonribosomal peptides [Pathway ID:ko01053]	-
**9.**	**Folding, sorting and degradation**	-	Protein export [Pathway ID:ko03060]
		-	Protein processing in endoplasmic reticulum [Pathway ID:ko04141]
		-	SNARE interactions in vesicular transport [Pathway ID:ko04130]
		-	RNA degradation [Pathway ID:ko03018]
**Down regulated process**			
**1.**	**Energy metabolism**	Photosynthesis [Pathway ID:ko00195]	-
**2.**	**Lipid metabolism**	-	Glycerophospholipid metabolism [PATH:ko00564]
**3.**	**Amino acid metabolism**	Arginine and Proline metabolism [Pathway ID:ko00330]	-
**4.**	**Glycan biosynthesis and metabolism**	-	Vitamin B6 metabolism [Pathway ID:ko00750]
**5.**	**Translation**	-	Protein processing in endoplasmic reticulum [Pathway ID:ko04141]
**6.**	**Signaling molecules and interaction**	-	ECM-receptor interaction [Pathway ID:ko04512]
**7.**	**Cellular Processes (Transport and Catabolism)**	Phagosome [Pathway ID:ko04145]	-

**Table 7:** List of DEGS commonly expressed in sandalwood (*S*. *album*).

Sl No.	CDS_Unigenes_Transcript	DEGs of *S*. *album*	log2Fold Change	p-val	Significance	Regulation
**1.**	CDS_9540_Uni_11613_Trans_66422	ARM20318.1ICE1	-1.60	0.04	No	Down
**2.**	CDS_13993_Uni_17451_Trans_85781	ARM20326.1RAP2-4-like protein [*S*. *album*]	-0.46	0.75	No	Down
**3.**	CDS_16279_Uni_20501_Trans_95760	ANQ46483.1,cytochrome P450 reductase [*S*. *album*]	-0.87	0.32	No	Down
**4.**	CDS_1770_Uni_2262_Trans_32293	ADO87007.1E, E-farnesyl diphosphate synthase/ AGV01244.1 farnesyl diphosphate synthase [*S*. *album*]	1.86	0.02	Yes	Up
**5.**	CDS_17842_Uni_22680_Trans_103059	ARM20329.1C3H29 [*S*. *album*]	0.28	0.76 0.73	No	Up
			0.31			
	CDS_17843_Uni_22681_Trans_103060					
**6.**	CDS_18136_Uni_23090_Trans_104382	ANQ46482.1cytochrome P450 reductase [*S*. *album*]	0.47	0.68 0.67 0.52	No	Up Down
			0.45			
	CDS_18138_Uni_23092_Trans_104384		-0.56			
	CDS_18143_Uni_23098_Trans_104395					
**7**	CDS_19033_Uni_24363_Trans_108464	ANQ46485.1cytochrome b5 [*S*. *album*]	0.79	0.21	No	Up
**8.**	CDS_19930_Uni_25598_Trans_112532	AHB33939.1bergamotene oxidase [*S*. *album*]	0.43	0.57 0.32 0.40 0.39	No	Up
			1.05			
	CDS_19932_Uni_25600_Trans_112537		1.00			
	CDS_19933_Uni_25601_Trans_112538		1.03			
	CDS_19934_Uni_25602_Trans_112543					
**9.**	CDS_19935_Uni_25603_Trans_112546	AHB33943.1CYP76F43 [*S*. *album*]	0.46	0.56	No	Up
**10.**	CDS_2184_Uni_2749_Trans_35028	ARM20319.1COR413-TM1 [*S*. *album*]	0.37	0.62 0.66	No	Up
			0.33			
	CDS_2185_Uni_2750_Trans_35029					
**11.**	CDS_22250_Uni_28800_Trans_123276	ADK89203.1cinnamyl alcohol dehydrogenase, partial [*S*. *album*]	1.18	0.08	No	Up
**12.**	CDS_23330_Uni_30495_Trans_128410	ANQ46486.1cytochrome b5 [*S*. *album*]	0.85	0.32	No	Up
**13.**	CDS_5419_Uni_6545_Trans_48603	ADO87008.1isopentyl diphosphate isomerase [*S*. *album*]	1.82	0.03	Yes	Up
**14.**	CDS_13993_Uni_17451_Trans_85781	ARM20326.1RAP2-4-like protein [*S*. *album*]	-0.46	0.74	No	Down

The transcriptional mining identified ten unigenes participated in sandalwood oil biosynthesis with the upregulated relative gene expression log2FC *viz*, **(i)** *Geranyl geranyl diphosphate synthase* (*SaGPS*) (FC; 3.54), **(ii)** *Geranyl diphosphate synthase* (*SaGGPS*) (2.6), **(iii)** *3-hydroxy-3-methylglutaryl-coenzyme A reductase* (*Sa*HMG-CoA) (1.32), **(iv)** *Sa1-Deoxy-D-xylulose-5-phosphate synthase* (*SaDXS*) (0.675), **(v)** *E-E*, *Farnesyl pyrophosphate synthase* (*SaE-E-FDS*) (3.21), **(vi)** *Cytochrome P450 synthase* (*SaCYP450*) (2.43) **(vii)** *Farnesyl pyrophosphate synthase* (*SaFPPS*) (1.86), **(viii)** *Phenylalanine ammonia lyase* (*Sa*PAL) (2.1) **(ix)** *Monoterpene synthase* (*SaMTPS*) (2.76), **(x)** *5-enolpyruvylshikimate 3-phosphate synthase* (*SaESPS*) (1.4). Transcripts encoding *SaFPPS* gene in *Sa*SHc showed 10 fold higher than *Sa*SLc accessions (Table 8 and S4 Table).

**Table 8:** Relative expression of high oil yielding (*Sa*SHc) genes, coding sequence, unigenes, transcripts, log2 fold change and regulation.

SI No.	Genic SSR primers	CDS	Unigenes	Transcripts	Log2fold change	Regulation
1.	Geranyl pyrophosphate synthase (*GPS*)	2915	3614	38599	2.61	Upregulation
2.	Geranyl geranyl pyrophosphate synthase (*GGPS*)	9544	11617	66429	3.54	Upregulation
3.	3-Hydroxy-3-methylglutaryl-CoA reductase (*HMG-CoA*)	11763	14481	75932	1.32	Upregulation
4.	1-Deoxy-D-xylulose5-phosphate synthase (*DXS*)	21435	27658	119568	0.67	Upregulation
5.	E, E, Farnesyl diphosphate synthase (*E-E-FDS*)	7514	29031	123929	2.65	Upregulation
6.	Cytochrome P450 synthase (*CYP450*)	6012	7205	5126	2.43	Upregulation
7.	Farnesyl pyrophosphate synthase (*FPPS*)	1770	2262	32293	1.86	Upregulation
8.	Phenylalanine ammonia lyase (*PAL*)	21850	28225	121398	3.55	Upregulation
9.	Monoterpene synthase (*MTPS*)	1948	2474	33744	2.98	Upregulation
10.	5-enolpyruvylshikimate 3-phosphate synthase (*ESPS*)	11286	13874	74066	2.17	Upregulation

### Transcription factors involved in sandalwood oil biosynthesis

Transcription factors are important regulators, which can regulate the development, maturation, oil biosynthesis and its accumulation in plants/trees. The RNA-sequence database of the current study revealed 47 and 41 families of transcription factors in high and low oil yielding sandalwood accessions. Some of the abundant transcription factors included *CDK7*, *ERCC2*, *ERCC3*, *CCNH*, *TAF8*, *TAF4*, *TFIIA*, *TFIIB*, *GTF2A*, *GTF2* and *TBP* (Table 9). Total fourteen upregulated transcription factors were identified with the variation in copy numbers in respective samples *viz*, (1) transcription initiation factors *TFIID subunit-*6, five folds in *Sa*SHc and four folds in *Sa*SLc (K03131, 0.86) (2) transcription initiation factor TFIID TATA-box-binding protein (K03120, 0.64) (3) transcription initiation factor TFIIA small subunit (K03123 FC 0.50) (4) transcription initiation factor *TFIIF* subunit α two copy (K03138, 0.44) (5) transcription initiation factor *TFIIH* subunit2 (K03142, 0.44), (6) *cyclin-dependent kinase-*7 three copy in *Sa*SLc and one copy in *Sa*SHc (K02202, 0.42) (7) *cyclin* H one copy in *Sa*SHc and two copy in *Sa*SLc (K06634, 0.42) (8) CDK-activating kinase assembly factor MAT1 two copy in *Sa*SLc and one copy present in *Sa*SHc sample (K10842, 0.42) (9) transcription initiation factor TFIID subunit11 (K03135, 0.31) (10) transcription initiation factor TFIIF *β* subunit (K03139, 0.34), (11) transcription initiation factor TFIID subunit2 (K03128, 0.35), (12) transcription initiation factor TFIIE subunit *α*, two copy in *Sa*SHc (K03136, 0.24), (13) transcription initiation factor *TFIIE subunit β* (K03137, 0.27) (14) transcription initiation factor *TFIID subunit-*9B (K03133, 0.18) (S5 Table). Nine genes were expressed downregulated with FC range from -578 to -0.63. It included (1) DNA excision repair protein *ERCC-3*, 2 copy (K10844, -0.75), (2) transcription initiation factor *TFIID* subunit1 (K03125, -0.57), (3) transcription initiation factor *TFIID* subunit4, two copy (K03129, -0.17), (4) transcription initiation factor *TFIID* subunit12 (K03126, -0.17), (5) Transcription initiation factor *TFII*-A large subunit three copy in in both the accessions (K03122–0.10), (6) transcription initiation factor *TFIIH* subunit 4 copy in *Sa*SHc (K03144, -1.0), (7) transcription initiation factor *TFIIH* subunit three copy in *Sa*SHc (K03143, -0.23), (8) transcription initiation factor *TFIIB* four copy in *Sa*SHc (K03124, -0.23), (9) transcription initiation factor *TFIID* subunit 5, two copy in *Sa*SHc and one copy present in *Sa*SLc sample (K03130–0.63) (Table 9).

**Table 9:** List of transcription factors and genes encoding key enzymes for sandalwood oil biosynthesis whose expressions were altered in high oil (*Sa*SHc) and low oil yielding (*Sa*SLc) sandalwood (*S*. *album*).

SI No.	Transcription factors (ID) (*Sa*SHc & *Sa*SLc)	Annotations
1.	TFIIA1, GTF2A1, TOA1 (K03122) (3&3)	Transcription initiation factor (TIF) TFIIA large subunit
2.	TFIIA2, GTF2A2, TOA2 (K03123) (1&1)	(TIF) TFIIA small subunit
3.	TFIIB, GTF2B, SUA7, tfb (K03124) (4&3)	(TIF) TFIIB
4.	TBP, tbp (K03120) (2&1)	(TIF) TFIID TATA-box-binding protein
5.	TAF1 (K03125) (1&1)	(TIF) TFIID subunit 1
6.	TAF2 (K03128) (1&1)	(TIF) TFIID subunit 2
7.	TAF8 (K14649) (2&1)	(TIF) TFIID subunit 8
8.	TAF5 (K03130) (2&2)	(TIF) TFIID subunit 5
9.	TAF4 (K03129) (2&2)	(TIF) TFIID subunit 4
10.	TAF12 (K03126) (1&1)	(TIF) TFIID subunit 12
11.	TAF6 (K03131) (5&4)	(TIF) TFIID subunit 6
12.	TAF9B, TAF9 (K03133) (1&1)	(TIF) TFIID subunit 9B
13.	TAF11 (K03135) (1&1)	(TIF) TFIID subunit 11
14.	TFIIE1, GTF2E1, TFA1, tfe (K03136) (1&1)	(TIF) TFIIE subunit alpha
15.	TFIIE2, GTF2E2, TFA2 (K03137) (1&1)	(TIF) TFIIE subunit beta
16.	TFIIF1, GTF2F1, TFG1 (K03138) (2&2)	(TIF) TFIIF subunit alpha
17.	TFIIH2, GTF2H2, SSL1 (K03142) (1&1)	TFIIH subunit 2
18.	TFIIF2, GTF2F2, TFG2 (K03139) (1&1)	(TIF) TFIIH subunit 2
19.	TFIIH3, GTF2H3, TFB4 (K03143) (1&1)	(TIF) TFIIH subunit 3
20.	TFIIH4, GTF2H4, TFB2 (K03144) (1&1)	(TIF)TFIIH subunit 4
21.	ERCC3, XPB (K10843) (1&1)	DNA excision repair protein ERCC-3
22.	ERCC2, XPD (K10844) (2&2)	DNA excision repair protein ERCC-2
23.	CDK7 (K02202) (4&3)	Cyclin-dependent kinase 7
24.	MNAT1 (K10842) (2&2)	CDK-activating kinase assembly factor MAT1
25.	CCNH (K06634) (3&2)	Cyclin H

### Phylogenetic analysis of identified cytochrome family in RNA-seq of *S*. *album*

Cytochrome P450 mono-oxygenases putatively involved in sandalwood oil biosynthesis (Diaz-Chavez et al. 2013 [18]). In order to phylogenetic analysis of cytochromes, BLAST was performed on pooled RNA-seq data and total 237 cytochrome genes (FC 6.87–0.234) were listed in which 84 cytochrome genes were observed with FC>1.0. Based on their structures, total nine groups of cytochrome gens were resulted **i.** *Cytochrome* b561 **ii.** *Cytochrome* P450 **iii.** *Cytochrome c oxidase* **iv.** *Cytochrome* P45076C2 **v.** *Cytochrome c oxidase* subunit1 **vi.** *NADH-cytochromeb5 reductase* **vii.** *SaCYP736A167* **viii.** *mitochondrial cytochrome b* and **ix.** *Cytochrome-*P450 *E-class* (S6 Table).

### Distribution of shared gene clusters across plant species

**Fig 6:**
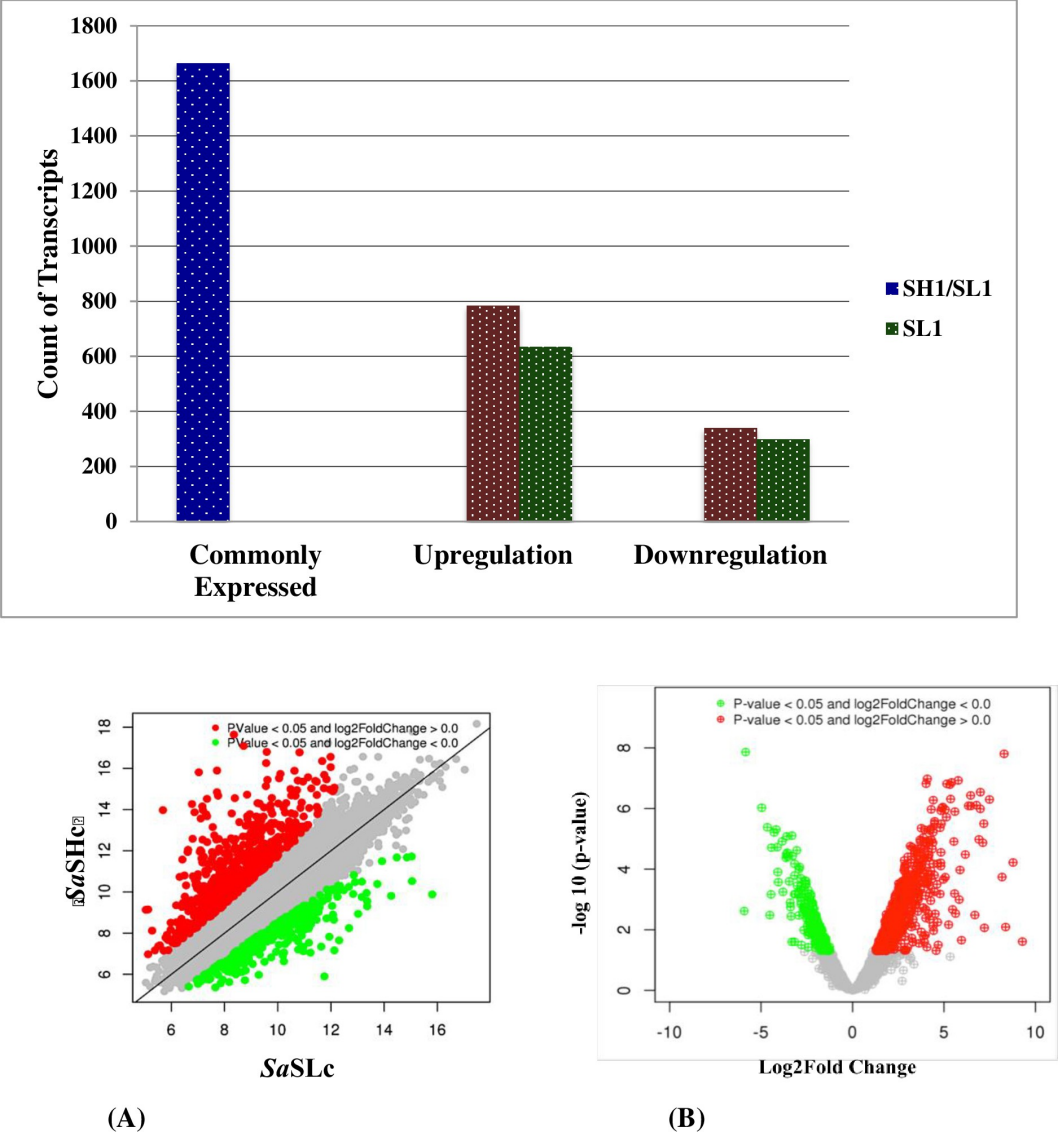
Top blast hit species distribution of coding sequence (CDS); Majority of the hits were found to be against *Vitis vinifera*.

In the current study, majority of the blast hits were found to be against *Vitis vinifera*, *Quercus suber*, *Juglans regia*, *Nelumbo nucifera*, *Thobroma cacao*, *Ziziphus jujuba*, *Hevea brasiliensis*, *Manihot esculenta* and *Jatropha curcus* (Fig 6). BLAST results were obtained for 91.77% of all the contigs with upregulated and downregulated genes (8.22% without BLAST hit). Whereby the 9 woody plant taxa *V*. *vinifera*: 4,710 (46.97%) *Q*. *suber*: 828 (8.25%), *J*. *regia*: 782 (7.82%), *N*. *nucifera*: 766 (7.64%), *T*. *cacao*: 460 (4.58%), *Z*. *jujuba*: 437 (4.35%), *H*. *brasiliensis*: 428 (4.26%), *M*. *esculenta*: 358 (3.57%), *J*. *curcus*: 338 (3.37%) and *A*. *thaliana* 23 (0.8%) with 896 genes were no blast hit were the species which gave the highest number of BLAST hits (S6 Fig). Although many numbers of transcripts were not functionally annotated, this study provides more than 20,842 annotated transcripts, which can be directly used for further research in sandalwood species. Total 784 genes were upregulated and BLAST results were obtained for 770 (98.2%) genes were shared clusters with other plant species and 41 (5.2%) was found no blast hit (S5 Fig). Total 339 genes were down regulated and BLAST results were obtained for 80.2% of all the contigs (19.2% without BLAST hit) (S6 Fig).

### Validation of the expression profiles of candidate genes involves in high oil biosynthesis of sandalwood by real time PCR (q-PCR)

**Fig 7:**
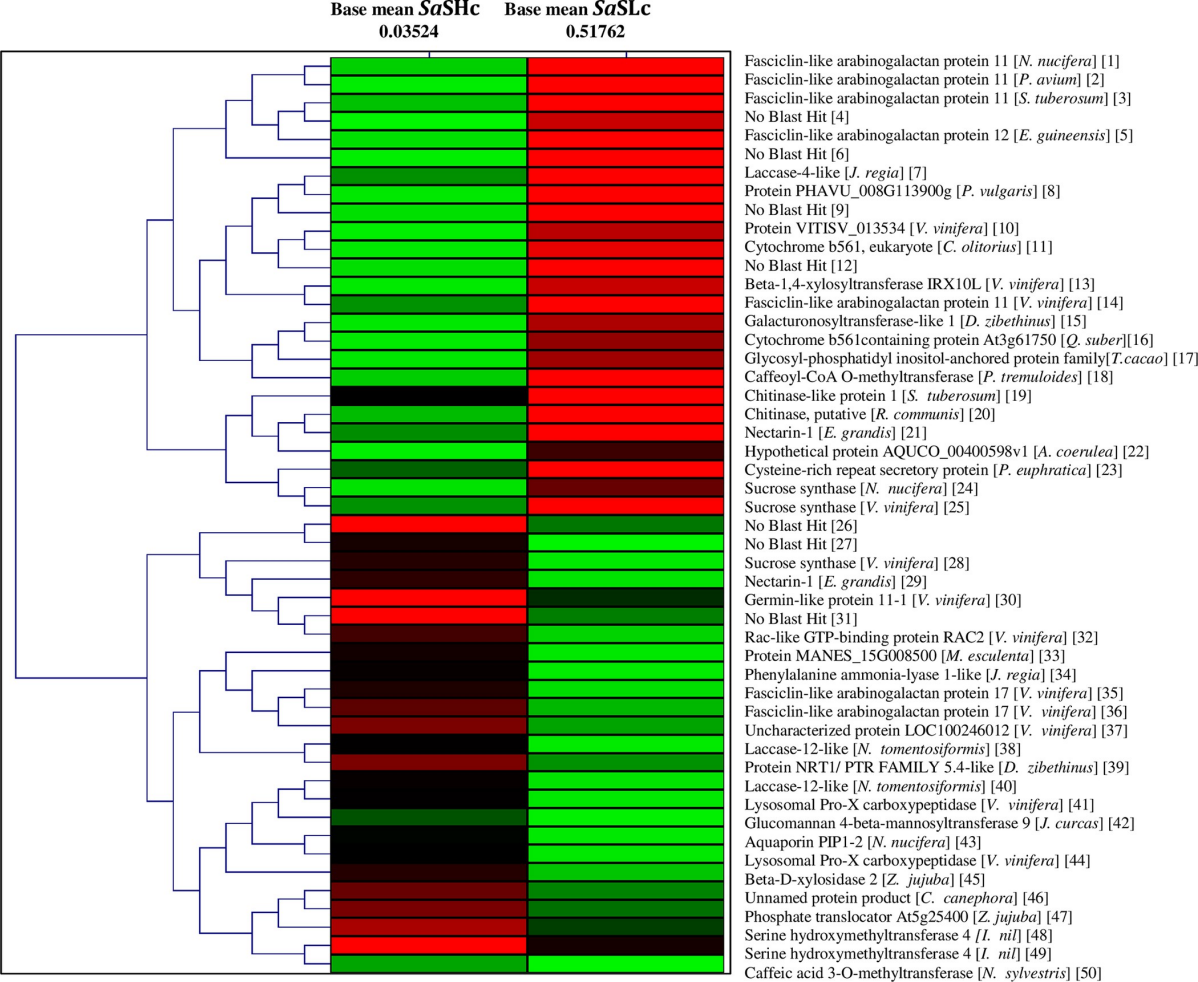
Validation of relative gene expression levels of differentially expressed genes by qRT-PCR. Purple and blue lines represent the RNA-Seq results, while red and green bars represent the qRT-PCR results. The error bars indicate the standard deviation.

To validate the expression profiles of candidate genes obtained from the RNA-Seq analysis, six candidate genes relate with oil biosynthesis in the transition zone of sandalwood were selected for qRT-PCR analysis. The expression levels of the selected genes were compared with RNA-seq results. The expression patterns of RNA-Seq and qRT-PCR revealed that the expression pattern of these genes were consistent which indicated the reliability of the RNA-seq data (Fig 7).

## Discussion

Sandalwood oil have a wide variety of uses including perfumery, pharmaceutical and toiletries, which makes understanding the regulation of high essential oil biosynthesis in sandalwood tree highly important [38, 39]. Due to unregulated harvesting many natural sources of elite sandalwood trees have been exhausted, in response to this sandalwood tree plantations have been established in many region of southern India. It has been reported that the oil content of sandalwood varies tree to tree with a negative correlation [8]. In this study we identified sandalwood trees of high and low oil content (*Sa*SHc and *Sa*SLc). Sandalwood oil biosynthesis and its regulation are extensively documented in sandalwood [18–20]. Our objective was to identify the transcriptomic responses considering high and low oil yield sandalwood grown in similar field condition. This study was focused on identifying genes involved in high oil biosynthesis and its regulation. In recent years, RNA-seq has been extensively employed for sandalwood oil biosynthesis pathway [18–20, 35]. To understand the dynamic regulation of oil accumulation and concentration variation, a comparative *De novo* transcriptome profiling of two identical accessions that differ significantly in oil content was carried out. Using this, we tried to infer the effect of change in gene structure difference in sandalwood accessions and underlying the molecular mechanism is important for developing high oil yielding cultivation of sandalwood (*Sa*SHc and *Sa*SLc). Various approaches for functional annotation of the assembled transcripts have been used to identify the genes in which mostly were involved in secondary metabolite biosynthesis in sandalwood. Functional annotation of assembled 6159 and 5,554 CDS of high and low oil yielding accessions of sandalwood showed high number of CDS were involved in carbohydrate metabolism followed by genetic information (Table 3). Based on the functional annotation enrichment analysis of the differentially expressed genes, identified some overrepresented genes participated in high oil biosynthesis with the highest 96.46% similarity in cytochrome b560 and Cytochrome b561 containing protein At3g61750 with 67.43%. It is generally accepted that identification of orthologous gene clusters helps in taxonomic and phylogenetic classification. We identified, 11,013 orthologous gene clusters, suggested their conservation in the ancestry. GO analysis of both accession showed that the majority of genes are enriched in molecular function and biological process (Table 2). Interestingly, a large fraction of genes involved in response to amino acid metabolism and transcription pathways (Table 4). A more number of unigenes were found to be involved in secondary metabolite biosynthesis in sandalwood was identified by mapping of unigenes against the KEGG pathway database (Table 4). Total number of unigenes including isoprenoid and putative terpenoid pathway genes were involved in the secondary metabolite biosynthesis. By comparing the transcriptome profiles of high and low oil yielding heartwood, high and low oil yielding heartwood, 31,918 unigenes were identified of DEGs (S3 Table). Among them the expression level of E-E Fernasyl diphosphate synthase/ Fernasyl diphosphate synthase in the terpenoid backbone biosynthesis was upregulated in the comparison (Table 8) indicating increased supply of precursors for diterpene biosynthesis. Other genes of interest emerged from this study, which include several members of the transcription factor family. 41 and 47 were all upregulated in the heartwood with high and low oil content accessions sandalwood (Table 9). In another study of sandalwood, 58 families of transcription factors were observed [21]. However, we were unable to detect some of the transcription factors in our data. The quantitative variations in the essential oil content in high and low oil yielding sandalwood accessions can be due to terpene synthase and other secondary metabolite related gene expression and regulation. The result of annotation indicated that more than 51% of assembled unigenes of sandalwood matched with the genomic database of other plants (Fig 6). The unigenes identified in this research, had a higher annotation percentage against the *Vitis vinifera* (25%) genome database compared to other plant database (Fig 6). KEGG analysis showed that the terpenoid synthesis and metabolism pathway was significantly enriched (Table 4) in sandalwood. A total 216 transcripts were involved in terpenoid biosynthesis. However, 181 transcripts were involved in low oil yielding sandalwood accession. Other transcriptome studies in sandalwood have detected low number of genes involved in oil biosynthesis [18–21, 35]. However no significant expression of genes directly involved in high oil biosynthesis was reported in Sandalwood. We identified selected candidate genes, which were specifically expressed in *Sa*SHc (Table 8) along with previously identified genes [18, 19, 21, 36]. The lower number presented in our data set is likely because core tissue of sandalwood were used for transcriptome analysis. The oil biosynthesis genes were abundantly expressed in *Sa*SHc when compared to *Sa*SLc accessions and validated the participation of genes in high oil biosynthesis Table 5. We observed *SaCYP736A*167 in our predicted gene sets, which identified as a candidate key oil biosynthesis gene in *S*. *album* in previous reports [18]. In this study identified a cohort of genes in the terpenoid backbone biosynthesis and monoterpenoid biosynthesis pathway that were commonly upregulated in heartwood of high oil content sandalwood compared to low oil content sandalwood. The knowledge obtained from this study could facilitate manipulation of sandalwood essential oil production through metabolic engineering of essential oil biosynthesis.

## Conclusion

The comparative analysis of the sandalwood oil accumulating core tissues of sandalwood showed that transcriptional regulation plays a key role in the considerable differences in oil content between high and low oil yielding sandalwood. To the best of our knowledge, this is the first study reporting the comparative transcriptomic response of sandalwood using RNA-Seq approach and identified different group of genes in high oil yielding samples under the similar condition. The present study generated a well-annotated pair end read RNA libraries and the results unveiled genome wide expression profile of sandalwood oil biosynthesis. Analysis of transcriptome data sets, identified transcripts that encode various transcription factor, metabolism of terpenoids, environment response element and biosynthesis of other secondary metabolites. Nevertheless, we also discovered some of the oil biosynthesis candidate genes SaCYP736A167, DXR, DSX and FPPS genes that participates in sandalwood oil biosynthesis and accumulation of oil in heartwood. The results suggested an intricate signalling and regulation cascade governing sandalwood oil biosynthesis involving multiple metabolic pathways. These findings have improved our understanding of the high sandalwood oil biosynthesis at the molecular level laid a solid basis for further functional characterization of those candidate genes associated with high sandalwood oil biosynthesis in *S*. *album*. Understanding the molecular mechanism of high and low oil sandalwood by RNA-seq will lead to significant information for farmers and forest department. The accessibility of a RNA-Seq for high oil yielding sandalwood accessions will have broader associations for the conservation and selection of superior elite samples/populations for further multiplications.

## Supporting information

S1 Table (DOCX)

S2 Table (DOCX)

S3 Table (DOCX)

S4 Table (DOCX)

S5 Table (DOCX)

S6 Table (DOCX)

S1 Fig (TIF)

S2 Fig (DOCX)

S3 Fig (DOCX)

S4 Fig (DOCX)

S5 Fig (DOCX)

S6 Fig (DOCX)
