# Supplementary material for: Comparative transcriptome profiling of high and low oil yielding Santalum album L
Source: PLoS One. 2022 Apr 28;17(4):e0252173. doi: 10.1371/journal.pone.0252173 (PMC9049570; doi:10.1371/journal.pone.0252173)
Supplement: S1 Table — (DOCX) [file pone.0252173.s001.docx]

| S1 Table. Morphological differentiation of the two contrasting Sandalwood (*S. album*) cores | | |
| --- | --- | --- |
|  | ***Sa*SHc** | ***Sa*SLc** |
| **Location** | Karnataka (India) | Karnataka (India) |
| **Age** | 15 years | 15 years |
| **Sample type** | High oil yielding accession | Low oil yielding accession |
| **Heartwood oil %** | 4.96 | 0.53 |
| **Oil quality (α & β-Santalol)** | 59.30 & 32.21 | 49.52 & 26.60 |
