# Supplementary material for: Comparative transcriptome profiling of high and low oil yielding Santalum album L
Source: PLoS One. 2022 Apr 28;17(4):e0252173. doi: 10.1371/journal.pone.0252173 (PMC9049570; doi:10.1371/journal.pone.0252173)
Supplement: S2 Table — (DOCX) [file pone.0252173.s002.docx]

| **S2 Table.** Primers used for qRT-PCR | | | | | |
| --- | --- | --- | --- | --- | --- |
| **Sl. No.** | **Primers** | **Forward** | **Reverse** | **Product size** | **Tm (^0^C)** |
|  | Geranyl pyrophosphate synthase (*Sa*GPS) | CGCCAGGTACACTTCCAAAT | AGGAGACATGTGGGGTGAAG | 158 bp | 60 |
|  | Geranyl geranyl pyrophosphate synthase (*Sa*GGPS) | GACGTAGGCATGGATCGTTT | AAGCGCTCTTGGAATCGTAA | 239 bp | 60 |
|  | 1-Deoxy-D-xylulose5-phosphate synthase (*Sa*DSX) | GCCTCAGCAACATCTTCACA | AGCGGAAATGCTTGTTATGG | 207 | 61 |
|  | Farnesyl pyrophosphate synthase (*Sa*FPPS) | TCGGAAAACCAAATTTCTCG | TGAGAGCCCTCGATTCAGTT | 151 | 61 |
|  | Monoterpene synthase (*Sa*MTPS) | ACATTGAAGCCCACAAAAGG | GATCTCGGAACGTCTTCAGC | 197 | 60 |
|  | Cytochrome P450 synthase  (*Sa*CYP450) | GTGGAAACAGGGGACTGAGA | CCGAATCCGAGTTCAGACAT | 217 | 61 |
