# Supplementary material for: Comparative transcriptome profiling of high and low oil yielding Santalum album L
Source: PLoS One. 2022 Apr 28;17(4):e0252173. doi: 10.1371/journal.pone.0252173 (PMC9049570; doi:10.1371/journal.pone.0252173)
Supplement: S3 Table — (DOCX) [file pone.0252173.s003.docx]

| **S3 Table.** Summary of annotations of assembled transcripts, unigenes, Coding Sequence (CDS), sample wise CDS and distribution of CDS in Sandalwood (*S. album*) | | | | | |
| --- | --- | --- | --- | --- | --- |
| **Transcripts** | | | | | |
| Number of Transcripts | 141,781 | | | | |
| Total transcript length (bases) | 16,285 billion | | | | |
| N50 | 2,044 | | | | |
| Maximum transcript length | 15,872 | | | | |
| Minimum transcript length | 201 | | | | |
| Mean transcript length | 1,149 | | | | |
| Number of unigenes | 31,918 | | | | |
| Total unigene length bases | 5.55 billion | | | | |
| N50 | 2,272 | | | | |
| Maximum unigene length | 1,587 | | | | |
| Minimum unigene length | 201 | | | | |
| Mean unigene length | 1,739 | | | | |
| **Unigenes** | | | | | |
| Number of unigenes | | | 31,918 | | |
| Total pooled unigene length bases (bp) | | | 5.55 billion | | |
| **Range of unigenes** | | | | | |
| 200$\leq$unigene $\leq$ 500 | | | 3,785 | | |
| 500$\leq$ unigene $\leq$ 1000 | | | 6,085 | | |
| 1000$\leq$ unigene $\leq$ 2000 | | | 11,582 | | |
| 2000$\leq$ unigene $\leq$ 3000 | | | 6,179 | | |
| 3000$\leq$ unigene $\leq$ 4000 | | | 2,688 | | |
| 4000$\leq$ unigene $\leq$ 5000 | | | 946 | | |
| $\geq$ 5000 | | | 653 | | |
| **Coding Sequences (CDS)** | | | | | |
| **Number of CDS** | | 2.271 million | | | |
| Total CDS length bases | | 2.810 billion | | | |
| Maximum CDS length | | 1.52 million | | | |
| Minimum CDS length | | 261 | | | |
| Mean CDS length | | 1,238 | | | |
| **Sample wise no. of CDS** | | ***Sa*SHc** 2.12 million | | ***Sa*SLc** 1.811 million | |
| Total CDS length (base) | | 2.657 billion | | 2.307 billion | |
| Maximum CDS length | | 1.164 million | | 1.527 million | |
| Minimum CDS length | | 264 | | 261 | |
| Mean CDS length | | 1,250 | | 1,247 | |
| **Distribution of pooled CDS** | | Total no. of CDS | | No. of CDS Blast Hit | No. of CDS without Blast Hit |
|  | | 22,710 | | 20,842 | 1,868 |
