# Supplementary material for: Comparative transcriptome profiling of high and low oil yielding Santalum album L
Source: PLoS One. 2022 Apr 28;17(4):e0252173. doi: 10.1371/journal.pone.0252173 (PMC9049570; doi:10.1371/journal.pone.0252173)
Supplement: S4 Table — (DOCX) [file pone.0252173.s004.docx]

| **S4 Table.** Details transcriptional mining identified transcripts, unigenes and CDS with log2 FC involved in Sandalwood oil biosynthesis | | | | |
| --- | --- | --- | --- | --- |
| **Sl No.** | **Transcript** | **Unigene** | **CDS** | **log2 fold change** |
| **(i) Geranyl Diphosphate Synthase (GPS)** | | | | |
|  | 126453 | 29829 | 22930 | 0.45 |
|  | 126598 | 29878 | 22954 | 0.73 |
|  | 126717 | 29900 | 22973 | 0.23 |
|  | 126733 | 29906 | 22978 | -0.74 |
|  | 35972 | 2987 | 2389 | 0.14 |
|  | 35976 | 2990 | 2392 | 0.18 |
| **(ii) Geranyl geranyl Diphosphate Synthase (GGPS)** | | | | |
|  | 66429 | 11617 | 9544 | 3.54 |
|  | 70570 | 12844 | 10488 | -0.01 |
|  | 75438 | 14333 | 11654 | 0.32 |
|  | 75439 | 14334 | 11655 | -0.03 |
|  | 87844 | 18072 | 14442 | -0.22 |
|  | 96039 | 20581 | 16344 | 0.53 |
|  | 38599 | 3614 | 2915 | 1.27 |
|  | 50243 | 6944 | 5775 | 0.26 |
|  | 51916 | 7356 | 6148 | 0.43 |
| **(iii) 3-Hydroxy-3-Methylglutaryl-coenzyme A reductase (HMG-CoA)** | | | | |
|  | 126626 | 29886_ | 22962 | -0.31 |
|  | 126631 | 29887 | 22963 | -0.47 |
|  | 75932 | 14481 | 11763 | 0.13 |
|  | 84436 | 17076 | 13730 | -0.12 |
| **(iv) D-xylulose-5-phosphate synthase (DXS)** | | | | |
|  | 65383 | 11280 | 9278 | 2.88 |
|  | 65382 | 11279 | 9277 | 2.84 |
|  | 65386 | 11282 | 9279 | -0.62 |
|  | 65387 | 11283 | 9280 | 2.83 |
|  | 65388 | 11284 | 9281 | 2.84 |
|  | 88610 | 18310 | 14626 | 1.18 |
|  | 88611 | 18311 | 14627 | 1.44 |
|  | 88612 | 18312 | 14628 | 1.38 |

| **S4 Table.** Details transcriptional mining identified Transcripts, unigenes and CDS with log2 FC involved in Sandalwood oil biosynthesis | | | | | |
| --- | --- | --- | --- | --- | --- |
| **(v) E, E, Farnesyl diphosphate synthase (E-E-FDS)** | | | | | |
| **Sl No.** | **Transcript** | **Unigene** | | **CDS** | **log2 fold change** |
| 1. | 123929 | 29031 | | 7514 | 1.23 |
|  |  |  | |  |  |
|  | 84691 | 17152 | | 13787 | 0.86 |
|  | 13310 | 900 | | 721 | 2.32 |
|  | 23001 | 1518 | | 1200 | 4.53 |
|  | 105953 | 23567 | | 18469 | 4.34 |
| **(vi) Farnesyl Pyrophosphate Synthase (FPPS)** | | | | | |
|  | 57837 | | 9030 | 7513 | 0.02 |
|  | 57846 | | 9031 | 7514 | -0.18 |
|  | 75177 | | 14255 | 11590 | 0.06 |
|  | 75182 | | 14258 | 11592 | 0.14 |
| 5. | 32293 | | 2262 | 1770 | 1.86 |
|  | 39264 | | 3827 | 3101 | 0.12 |
|  | 39265 | | 3828 | 3102 | 0.25 |
|  | 39267 | | 3829 | 3103 | 0.33 |
|  | 57837 | | 9030 | 7513 | 0.02 |
|  | 57846 | | 9031 | 7514 | -0.12 |
| **(vii) Cytochrome P450 Synthase (CYP-450)** | | | | | |
| 1. | 93250 | | 1796 | 2296 | 4.34 |
| 2. | 3672 | | 41475 | 4512 | 6.46 |
| 3. | 126208 | | 29757 | 22873 | 0.45 |
| 4. | 103051 | | 17840 | 22676 | 3.89 |
| 5. | 65195 | | 11226 | 9230 | 4.56 |
| **(viii) Phenylalanine Ammonia Lyase (PAL)** | | | | | |
|  | 121398 | | 28225 | 21850 | 5.07 |
|  | 121399 | | 28226 | 21851 | 4.79 |
|  | 121401 | | 28228 | 21853 | 0.03 |
|  | 121407 | | 28229 | 21854 | 4.33 |
| **(ix) 5-Enolpyruvylshikimate 3-phosphate Synthase (ESPS)** | | | | | |
|  | 74066 | | 13874 | 11286 | 2.38 |
|  | 74069 | | 13877 | 11289 | 2.48 |
|  | 103037 | | 22672 | 17836 | 2.23 |
|  | 103044 | | 22674 | 17838 | 1.83 |
|  | 103051 | | 22676 | 17840 | 1.93 |
| **(x) Monoterpene Synthase (MTPS)** | | | | | |
|  | 33744 | | 2474 | 1948 | 2.76 |
|  | 65185 | | 11221 | 9226 | 2.84 |
|  | 49408 | | 6753 | 5607 | 3.98 |
|  | 65348 | | 11266 | 9264 | 2.34 |
