# Supplementary material for: Comparative transcriptome profiling of high and low oil yielding Santalum album L
Source: PLoS One. 2022 Apr 28;17(4):e0252173. doi: 10.1371/journal.pone.0252173 (PMC9049570; doi:10.1371/journal.pone.0252173)
Supplement: S5 Table — (DOCX) [file pone.0252173.s005.docx]

| **S5 Table.** Comparative analysis of representative up and down regulated transcription factors in high oil (*Sa*SHc) and low oil (*Sa*SHc) yielding Sandalwood (*S. album*) | | | | | | |
| --- | --- | --- | --- | --- | --- | --- |
| **SI No.** | **Transcription factors** | *Sa*SHc | *Sa*SHc | **KO IDs** | **log2 Fold Change** | **p-value** |
|  | Transcription initiation factor TFIIA large subunit | 3 | 3 | K03122 | -0.10 | 0.54 |
|  | Transcription initiation factor TFIIA small subunit | 1 | 1 | K03123 | 0.50 | 0.48 |
|  | Transcription initiation factor TFIIB | 4 | 3 | K03124 | -0.23 | 0.78 |
|  | Transcription initiation factor TFIID TATA-box-binding protein | 2 | 1 | K03120 | 0.646 | 0.38 |
|  | Transcription initiation factor TFIID subunit 1 | 1 | 1 | K03125 | -0.57 | 0.50 |
|  | Transcription initiation factor TFIID subunit 2 | 1 | 1 | K03128 | 0.35 | 0.58 |
|  | Transcription initiation factor TFIID subunit 8 | 2 | 1 | K14649 | 0.43 | 0.59 |
|  | Transcription initiation factor TFIID subunit 5 | 2 | 2 | K03130 | -0.63 | 0.42 |
|  | Transcription initiation factor TFIID subunit 4 | 7 | 2 | K03129 | -0.17 | 0.80 |
|  | Transcription initiation factor TFIID subunit 12 | 4 | 1 | K03126 | -0.17 | 0.82 |
|  | Transcription initiation factor TFIID subunit 6 | 5 | 4 | (K03131 | 0.86 | 0.53 |

| **S5 Table.** Comparative analysis of representative up and down regulated transcription factors in high oil (*Sa*SHc) and low oil ( *Sa*SHc) yielding Sandalwood (*S. album*) | | | | | | |
| --- | --- | --- | --- | --- | --- | --- |
| **SI No.** | **Transcription factors** | **SH1** | **SL1** | **KO IDs** | **log2 Fold change** | **p-value** |
| 12. | Transcription initiation factor TFIID subunit 9B | 2 | 1 | K03133 | 0.1811 | 0.80 |
| 13. | Transcription initiation factor TFIID subunit 11 | 3 | 0 | K03135 | 0.35 | 0.66 |
| 14. | Transcription initiation factor TFIIE subunit alpha | 2 | 1 | K03136 | 0.24 | 0.77 |
| 15. | Transcription initiation factor TFIIF subunit alpha | 2 | 2 | K03138 | 0.44 | 0.54 |
| 16. | Transcription initiation factor TFIIH subunit 2 | 1 | 1 | K03142 | 6.16 | 0.32 |
| 17. | Transcription initiation factor TFIIH subunit 4 | 2 | 1 | K03144 | -1.04 | 0.13 |
| 18. | DNA excision repair protein ERCC-2 | 4 | 2 | K10844 | -0.75 | 0.25 |
| 19. | DNA excision repair protein ERCC-3 | 1 | 1 | K10843 | -0.23 | 0.79 |
| 20. | Cyclin-dependent kinase 7 | 4 | 3 | K02202 | 0.95 | 0.25 |
| 21. | CDK-activating kinase assembly factor MAT1 | 4 | 1 | K10842 | 0.42 | 0.51 |
| 22. | Cyclin H | 3 | 2 | K06634 | 0.42 | 0.65 |
