# Supplementary material for: Comparative transcriptome profiling of high and low oil yielding Santalum album L
Source: PLoS One. 2022 Apr 28;17(4):e0252173. doi: 10.1371/journal.pone.0252173 (PMC9049570; doi:10.1371/journal.pone.0252173)
Supplement: S6 Table — (DOCX) [file pone.0252173.s006.docx]

| **S6 Table.** Nine different cytochrome gene family identified in high oil (*Sa*SHc) and low oil (*Sa*SHc) yielding Sandalwood (*S. album*) | | |
| --- | --- | --- |
| **Sl No.** | **Cytochromes** | **Log2 fold change** |
|  | Cytochrome b561 | 7.0 |
|  | Cytochrome P450 | 3.0 |
|  | Cytochrome c oxidase | 3.0 |
|  | Cytochrome P45076C2 | 2.5 |
|  | Cytochrome c oxidase subunit 1 | 2.9 |
|  | NADH-cytochromeb5 redutase | 4.9 |
|  | SaCYP736A167 | 6.8 |
|  | Cytochromeb mitochondrial | 2.4 |
|  | CytochromeP450 E-class | 2.8 |
