# Supplementary material for: Comparative transcriptome profiling of high and low oil yielding Santalum album L
Source: PLoS One. 2022 Apr 28;17(4):e0252173. doi: 10.1371/journal.pone.0252173 (PMC9049570; doi:10.1371/journal.pone.0252173)
Supplement: S6 Fig — (DOCX) [file pone.0252173.s012.docx]

**S6 Fig.** Distribution of downregulated shared gene clusters across plant species
